# Left-right symmetry breaking in mice by left-right dynein may occur via a biased chromatid segregation mechanism, without directly involving the *Nodal* gene

**DOI:** 10.3389/fonc.2012.00166

**Published:** 2012-11-16

**Authors:** Stephan Sauer, Amar J. S. Klar

**Affiliations:** Gene Regulation and Chromosome Biology Laboratory, Frederick National Laboratory for Cancer Research, FrederickMD, USA

**Keywords:** laterality development, left-right dynein, asymmetric cell division, DNA strands differentiation, selective chromatid segregation

## Abstract

Ever since cloning the classic *iv* (*inversed* *viscerum*) mutation identified the “*left-right dynein*” (*lrd*) gene in mice, most research on body laterality determination has focused on its function in motile cilia at the node embryonic organizer. This model is attractive, as it links chirality of cilia architecture to asymmetry development. However, *lrd* is also expressed in blastocysts and embryonic stem cells, where it was shown to bias the segregation of recombined sister chromatids away from each other in mitosis. These data suggested that *lrd* is part of a cellular mechanism that recognizes and selectively segregates sister chromatids based on their replication history: old “Watson” versus old “Crick” strands. We previously proposed that the mouse left-right axis is established via an asymmetric cell division prior to/or during gastrulation. In this model, left-right dynein selectively segregates epigenetically differentiated sister chromatids harboring a hypothetical “*left-right axis development 1*” (“*lra1*”) gene during the left-right axis establishing cell division. Here, asymmetry development would be ultimately governed by the chirality of the cytoskeleton and the DNA molecule. Our model predicts that randomization of chromatid segregation in *lrd* mutants should produce embryos with 25% situs solitus, 25% situs inversus, and 50% embryonic death due to heterotaxia and isomerism. Here we confirmed this prediction by using two distinct *lrd* mutant alleles. Other than *lrd*, thus far *Nodal* gene is the most upstream function implicated in visceral organs laterality determination. We next tested whether the *Nodal* gene constitutes the *lra1* gene hypothesized in the model by testing mutant’s effect on 50% embryonic lethality observed in *lrd* mutants. Since *Nodal* mutation did not suppress lethality, we conclude that *Nodal* is not equivalent to the *lra1* gene. In summary, we describe the origin of 50% lethality in *lrd* mutant mice not yet explained by any other laterality-generating hypothesis.

## INTRODUCTION

**FIGURE 1:**
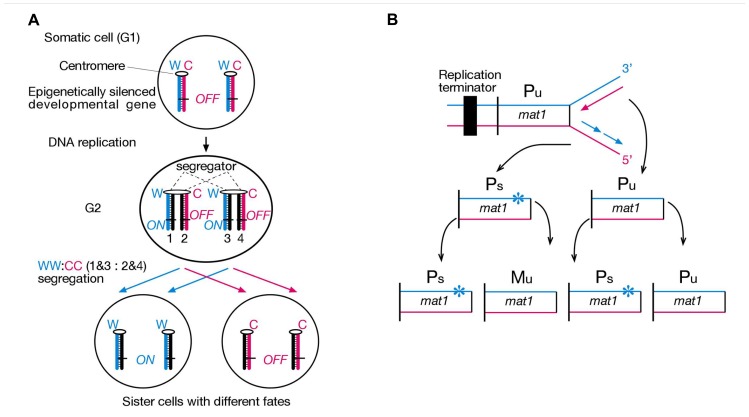
**Strand-specific imprinting in diploid and haploid organisms**. **(A)** Hypothetical asymmetric cell division according to our strand-specific imprinting and selective segregation (SSIS) model. Only one pair of homologous chromosomes is illustrated. Lagging versus leading strand DNA replication epigenetically differentiates an important developmental gene on sister chromatids, *ON* in one and *OFF* in the other. A segregator, such as left-right dynein, “sorts” sister centomeres/chromatids according to their replication history in G2, causing selective segregation of older Watson template strands into specific daughter cell, and older Crick template strands into the other daughter cell (named WW:CC segregation). Hence, asymmetric DNA replication-coupled epigenetic chromatin modification and selective sister chromatid segregation in the parent cell can specify different developmental potentials to daughter cells (Klar, 1994). Symbols: W, template “Watson” strand, C, template “Crick” strand. Numbers 1–4 represent specific chromatids with respect to their strands’ constitution. **(B)** Illustration of how lagging strand-specific imprinting explains the “1 in 4 granddaughters switching” rule in *S. pombe* mating-type switching. The *mat1* locus efficiently switches P and M mating-type gene information by a cell cycle controlled DNA transposition mechanism. A replication terminator ensures unidirectional DNA replication of the *mat1* locus, and lagging strand DNA synthesis installs an imprint (indicated by star) in a sequence- and strand-specific manner in an unswitcable (Pu) cell. The imprint confers competence for switching at the *mat1* locus only in the daughter cell inheriting the imprinted chromosome (Ps), which transposes opposite mating-type information copied from the silenced donor loci into the *mat1* locus only in one of the sister chromatids (Klar, 2007).

It is crucial for multicellular development that cells possess a memory system, which ensures stable inheritance of acquired developmental states during development of tissues and organs of an organism. The field of epigenetics studies this cellular memory system, and “epigenetic” is often defined as “mitotically heritable changes in gene expression that do not involve modulation of the primary DNA sequence.” For development, it is equally important that cells are able to change their acquired developmental state and differentiate along evolutionarily defined lineage paths. A crucial question is how epigenetic information can be changed and passed onto developmentally differentiated sister cells during asymmetric cell division. We proposed a solution to this problem. Namely, sister chromatids can be epigenetically differentiated regarding a developmentally important gene during S-Phase, based on lagging versus leading strand DNA replication, followed by selective sister chromatid segregation to specific daughter cells (**Figure 1A**). Our Somatic Strand-specific Imprinting and selective sister chromatid Segregation (SSIS) model (Klar, 1994) postulates that a specific daughter inherits both template Watson and first time synthesized Crick strand-containing (WC’) homologous chromosomes, thereby the other daughter inherits with both new Watson and old Crick (W’C) homologous chromosomes (referred to as WW:CC segregation pattern). As a consequence, a single gene or a gene cluster is poised for expression in one daughter cell and silenced in the other daughter cell. Likewise, if sister chromatids were selectively segregated in a WC:WC fashion, then both daughter cells would inherit equivalent epigenetic make ups and hence retain similar developmental potentials, as seen in symmetrical stem cell divisions. The SSIS model is based on studies on fission yeast (*Schizosaccharomyces pombe*) mating-type switching (Klar, 2007), and has been tested *in vitro* in mouse embryonic stem (ES) cells (Armakolas and Klar, 2006,^2007^), and *in vivo* in a mouse model for body laterality development (this study).

*Schizosaccharomyces pombe* is a haploid unicellular eukaryote, whose cells either express P or M mating-type information from the alternate alleles of the *mat1* locus residing in chromosome 2. The *mat1* mating-type content switches between M and P information by a cell cycle controlled DNA transposition mechanism, such that one out of four granddaughter cells switches cell type and expresses the mating-type opposite to that of the grandmother cell (**Figure 1B**). Genetic and biochemical analysis revealed that mating-type switching is controlled by lagging- versus leading-strand DNA replication at the *mat1* locus. In particular, lagging-strand DNA synthesis installs an imprint at *mat1* (most probably a two nucleotide long DNA:RNA hybrid from an incompletely removed Okazaki fragment), which initiates a double-strand break during the following S-Phase to start the DNA transposition event that underlies *mat1* switching. Hence developmental asymmetry between sister cells can be traced back to double helical structure of the *mat1* gene and lagging- versus leading-strand synthesis of specific DNA strands in two consecutive cell divisions (Klar, 2007).

We proposed that a similar mechanism might produce asymmetric cell divisions in diploid organisms by epigenetic means as well. First, strand-specific imprinting would epigenetically differentiate sister chromatids in S-Phase, and selective segregation of thus differentiated sister chromatids would create sister cells with different developmental fates. This model is called SSIS, and was initially developed by us to explain internal organ laterality development in vertebrates (Klar, 1994).

The development of bilateral asymmetry can be conceptually divided into three steps: First comes the initial symmetry-breaking event, usually ascribed to cellular amplification of a molecular chirality. This is followed by differential gene expression in cell fields on either side of the midline, which translates to step three, left/right (L/R) asymmetric organogenesis (Aw and Levin, 2009). For internal organ situs development in vertebrates, a great deal of molecular understanding has been achieved to decipher steps two and three, where many molecular pathways, seemingly conserved between model organisms, have been defined and well accepted (Nakamura and Hamada, 2012). For example, the TGF-β related signaling molecule Nodal is conserved in all deuterostomes examined, and usually specifies the left body-side (Chea et al., 2005). Its activity is inhibited toward the midline by Nodal’s own transcriptional targets of the Lefty family of diffusible molecules, which represents a prime example of a reaction-diffusion mechanism (Nakamura et al., 2006; Muller et al., 2012). In contrast, identity of the symmetry-breaking event, the “first event,” that initiates left-biased Nodal expression is controversial, because no unifying mechanism between vertebrate phyla has been in identified to date (Vandenberg and Levin, 2009). Some vertebrates such as mice, frogs, and zebrafish are proposed to employ motile cilia during gastrulation at equivalent embryonic organizer regions, known as the node, gastrocoel roof plate, and Kupffer vesicle, respectively. Cilia’s beating is thought to either transport a morphogen leftwards in extraembryonic space (Nonaka et al., 1998), or induce asymmetrical calcium signaling in conjunction with mechanosensory cilia (McGrath et al., 2003). As a consequence, Nodal signaling is induced more strongly in left-sided neighboring tissues (lateral plate mesoderm in the mouse), and its autoregulatory feedback loop with Lefty molecules confers robustness to the signaling cascade (Nakamura and Hamada, 2012). This model is very attractive as it links the molecular chirality of the cilium and its building blocks to chirality of the developing embryo. However, several observations prominently question this model’s universality, and some data would rather support a role for nodal cilia during step two of bilateral asymmetry development, namely, asymmetric gene expression on either side of the midline. First, pigs, for example, undergo L/R axis development without motile nodal cilia, undermining a universal role for motile cilia in vertebrate and mammalian symmetry-breaking (Vandenberg and Levin, 2010). Second, in species that employ cilia, a number of genes that are required for proper nodal cilia motility and positioning are also expressed in non-ciliated cells at much earlier embryonic stages. Examples include planar cell polarity genes *Vangl2* and *Dvl2*, *inversin* and *left-right dynein* (Aw and Levin, 2009). Thus, it is unclear whether these proteins exert their critical function in L/R axis development at the node. Third, mouse blastomere cells rearrangement has been shown to influence direction of embryonic turning, indicating that some aspects of laterality development certainly occur prior to gastrulation, and are independent of nodal cilia (Gardner, 2010). Last, both zebrafish and mouse mutants have been isolated, which show Kupffer vesicle or node ciliary defects but no L/R phenotypes, and *vice versa* (Vandenberg and Levin, 2010). Therefore, despite overwhelming evidence suggesting that cilia do have an important function in L/R asymmetry development in several species, they are unlikely to truly control initial symmetry-breaking in the embryo to generate L/R asymmetry (Tabin, 2005; Klar, 2008; Lobikin et al., 2012).

In 1959, Hummel and Chapman (1959) first described the recessive *iv* (*inversed* viscerum) mutation, where 50% of homozygous mice develop situs inversus (i.e., mirror-image reversal of internal organs), and 50% have normal organ situs. Parental organ situs does not affect organ situs of the offspring, thus this mutation randomizes L/R asymmetry. More detailed analysis revealed high rates of heterotaxia (random and independent sidedness of internal organs) affecting both normal and situs inversus homozygous mutants at similar rates and severity (Layton, 1978). This suggests that in addition to its involvement in the first step of asymmetry development, the *iv* gene product is also needed in the second and/or third conceptual steps described above. Molecular cloning by Supp et al. (1997) showed that the *iv* mutation changed a highly conserved glutamic acid to lysine within the motor domain of a dynein heavy chain gene, which was thereafter named *left-right dynein* (*lrd*). *Lrd* message was detected in blastocysts and (blastocyst-derived) ES cells, ventral node cells, and some ciliated embryonic and adult epithelia. It was classified as an axonemal dynein despite its obvious expression in many non-ciliated cell types. At the time the authors were not aware that node cells contain motile cilia, and even concluded that: ”...embryonic expression indicates that mechanisms other than ciliary movement are involved in L/R specification” (Supp et al., 1997). Later it was found that Node cells contained ciliated cells (Nonaka et al., 1998) whose motility is dependent on *lrd* (Supp et al., 1999). Technically difficult studies further showed that beating nodal cilia created a leftward fluid-flow in extraembryonic space (Nonaka et al., 1998), and artificial fluid-flow reversal had a dominant effect on situs development in normal and *lrd* mutant embryos (Nonaka et al., 2002). These data clearly highlight the node’s function as an embryonic organizer during L/R axis development. Whether the L/R asymmetry is truly established by nodal flow or whether this simply represents a “back-up” mechanism remains to be determined. To address this question, our lab has started to generate a conditional allele for *lrd* to discriminate between early cytoplasmic and later axonemal (cilia) functions.

A study from our lab has provided genetic evidence that *lrd* does indeed have a functional role in non-ciliated cells (Armakolas and Klar, 2007). Liu et al. (2002) had engineered mouse ES cells lines, which allowed for selection of Cre/loxP-mediated mitotic recombinants between homologs. If recombination happens in G2, recombined chromatids can either segregate together (Z segregation) or into different sister cells (X segregation). X segregants thereby acquire homozygosity of any heterozygous marker distal to the crossover site. Interestingly, centromere-proximal loxP sites on chromosome 7 (DT1E9 cell line) always led to X segregation, whereas loxP sites on chromosome 11 or further centromere-distal on chromosome 7, produced the usually expected random mix of X and Z segregants (Liu et al., 2002). Differentiation of DT1E9 ES cells to endoderm cells preserved the exclusive X segregation pattern, whereas neuroectoderm cells showed exclusive Z segregation. Three other *in vitro* differentiated cell types showed random patterns (Armakolas and Klar, 2006). We proposed that cell type-specific biased segregation patterns were due to selective chromatid recombination as well as selective segregation of chromosome 7 sister chromatids in mitosis. Remarkably, *lrd* mRNA expression was evident in ES, endoderm, and neuroectoderm cells, and RNAi-mediated knockdown randomized segregation patterns, consistent with our SSIS model (Klar, 2008). In this model, *lrd* would “sort” sister chromatids based on their replication history and selectively segregate sister centromeres to sister cells (**Figure 1A**). We propose that *lrd*’s function in non-ciliated cells is to bias sister chromatid segregation of one or a specific set of chromosomes. By theory, this function is not confined to a single L/R axis establishing asymmetric cell division, but probably happens in other developmental contexts where asymmetric or strictly symmetric cell divisions occur. Additional support for this is provided by *lrd*’s expression profile available on the gene annotation portal biogps.org, where *lrd* shows high expression in hematopoietic stem cells (http://www.biogps.org/#goto). Here we tested developmental biology predictions of the SSIS model concerning the *lrd* mutant. In a second experiment we tested whether the *Nodal* gene comprises the “*left-right axis development 1*” (*lra1*) gene specified in the SSIS model.

## MATERIALS AND METHODS

### MOUSE BREEDING AND HUSBANDRY

*Lrd*-Neo-GFP mice were a kind gift from Dr. Martina Brueckner at Yale University, New Haven, CT. The iv stock (EM:02531) was purchased (live) from EMMA repository, Harwell, UK. Delta *Nodal* mice were a kind gift from Dr. Michael Kuehn, Frederick National Laboratory, MD. All mice were kept according to Animal Care and User Committee (ACUC) guidelines, Frederick National Laboratory, MD.

### GENOTYPING

Between 3 and 4 weeks of age, tailclips were performed according to ACUC guidelines. Tails were digested by overnight incubation at 55°C in 200 µl of tail buffer [100 mM NaCl, 10 mM Tris-HCl pH 7.5, 10 mM EDTA, 0.5% (w/v) *N*-Lauroylsarcosine, 100 µg/ml Proteinase K]. The solution was then diluted 1:1 with dH_2_O, 1 µl was used for PCR reactions. *Lrd*-Neo-GFP primers: wtaF3: CTCTGCAGGCAGAGCGGCT, taR3: GCTTGCCGGTGGTGCAGA, wtR3: CGGGTCTAGGGCAAAGCGTT. PCR: 95°C 2 min – 34× (94°C 20 s, –64.5°C 20 s, –72°C 30 s) 72°C 5 min. wt allele: 194 bp, targeted allele: 266 bp. *Nodal* Delta primers: F4299: CAGAAGAG-GGATTTGGGGTTTGCAG, R4457: GATCGGAACTCAGGAACCTAGAAAC. 95°C 2 min – 32× (94°C 30 s, – 65°C 30 s, –72°C 30 s) 72°C 5 min. Targeted (delta) allele: ~180 bp. iv primers: 1959 TaqaI F: GCTAACCACCAACCACATGCTG, 1959 TaqaI R: CACGGATTCCAGCCCAGATC. 25 µl PCR product was digested with 25 U of Taq alpha I (NEB) in a 40 µl reaction, at 65°C for 45 min. The iv mutation destroys the Taq alpha I site in the PCR fragment. wt bands: 92 bp, iv band: 184 bp.

## RESULTS

### A TEST OF A KEY PREDICTION OF THE SSIS MODEL

**FIGURE 2:**
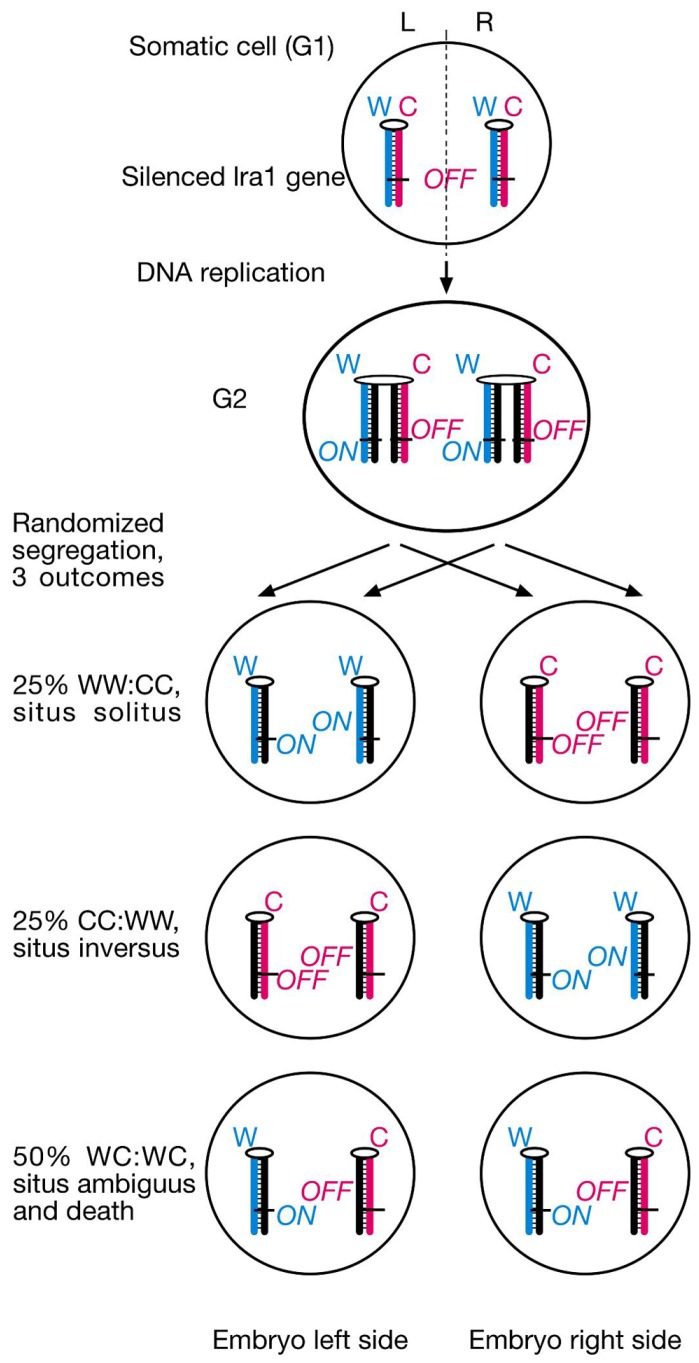
**SSIS-predictions concerning embryo situs and survival rates of *lrd* mutants**. Proposed laterality-generating asymmetric cell division is randomized in the *lrd* mutant. The future L/R axis is set by cytoplasmic polarization and alignment of a single cell with respect to the anterior-posterior and dorsal-ventral body axes. Sister chromatids containing a hypothetical “leftness-encoding” left-right axis-establishing gene 1 (*lra1*) are epigenetically differentiated. Normally, left-right dynein would selectively segregate older Watson template strand-containing sister chromatids harboring *lra1* “*ON*” epialleles to the left body side, and older Crick template strand-containing sister chromatids harboring *lra1* “*OFF*” epialleles to the right body side as described in Figure 1A. Randomized segregation due to left-right dynein mutation will result in three different outcomes shown here: 25% WW:CC cell pairs, causing normal situs development, 25% CC:WW cell pairs, causing development of situs inversus, and 50% WC:WC cell pairs, causing severe developmental situs abnormalities incompatible with survival.

Our model makes several testable predictions for the phenotype of the *lrd* mouse mutant. First, randomization of sister chromatid segregation during the critical L/R axis establishing cell division should have three different outcomes: 25% WW:CC cell pairs leading to normal organ situs later in development, 25% CC:WW cell pairs leading to inversed organ situs, and 50% WC:WC cell pairs causing embryonic lethality or death soon after birth due to isomerism (mirror-image sidedness of organs) or heterotaxia (random and independent sidedness of organs; **Figure 2**). Lethality occurs because of the *lra1* gene’s *ON/OFF* epiallele constitution in both sister cells. Prediction of 50% lethality in *lrd* mutant mice is a major difference between SSIS hypothesis and mainstream nodal cilia hypotheses for L/R axis development (Klar, 2008).

We acquired two different *lrd *mutant mouse strains, the original *iv* strain from EMMA repository and the *Lrd*-Neo-GFP mouse from Dr. Martina Brueckner’s laboratory (McGrath et al., 2003). The *iv* strain originated from a complex mixed background until siblings were inbred for >20 generations (EMMA repository, personal communication). The *Lrd-Neo-GFP* allele was introduced into ES cells of Sv129 genetic background (McGrath et al., 2003). To reduce background specific influences, both strains were bred onto C57BL/6 strain for one generation. Lethality rates were determined at weaning age (3–4 weeks) by PCR based genotyping of tailclip DNA (see section Materials and Methods).

*Lrd*-Neo-GFP mice carry a *GFP-lrd exon 1* fusion as well as a *Neo* cassette on the opposite strand of *lrd intron 1*. Since the *Neo* transgene is under the control of a very strong promoter and transcribed antisense to *lrd*, *lrd* transcription is effectively shut down and homozygous mutant mice are indistinguishable from true knockout mice: 50% of live animals exhibit situs inversus (McGrath et al., 2003). Several heterozygous intercrosses (*lrd*^+/−^) were set up and DNA from tails from 165 offspring was analyzed (**Table 1**). We detected 53 *lrd*^+/+^ : 90 *lrd*^+/−^ : 22 *lrd*^−/−^ animals. The SSIS hypothesis predicts ~24 (165/7) of live-born mice to be *lrd*^−/−^. This is because 1/8 (half of 1/4 animals with ^−/−^ genotype) of the initial number of homozygous mutant mice is expected to live, 1/8 is expected to die and thus reduce the total number of mice that are available for analysis to 7/8. As a result, 1/8 of the initial mice correspond to 1/7 of observable mice. If lethality was not an issue, then ~41 mice (1/4 of 165) should have the *lrd*^−/−^ genotype. Our observed number of 22 *lrd*^−/−^ mice is statistically equivalent to the SSIS-predicted number of 23.57 (*p*-value of ~0.6, chi-square test).

**Table 1:** Observed rates of allele frequencies: *Lrd-Neo-GFP *allele, *lrd*^+/−^ × *lrd*^+/−^.

*Lrd*^+/+^ (%)	*lrd*^+/−^ (%)	*lrd*^−/−^ (%)
53 (32)	90 (55)	22 (13)

Encouraged by the heterozygous cross results, we set up four *iv*^+/−^ X* iv*^−/−^ crosses. The results are summarized in **Table 2**. Conventionally 1/2 of the offspring is expected to be *iv*^−/−^. However if lethality affected 50% of the *iv*^−/−^ mice, this fraction would be reduced to 1/3 among live animals. Analysis of 111 offspring revealed 74 *iv*^+/−^ and 37 *iv*^−/−^ mice, which meets SSIS prediction exactly.

**Table 2:** Observed rates of allele frequencies: *iv *allele, *iv*^+/−^ × *iv*^−/−^.

*iv*^+/−^(%)	*iv*^−/−^ (%)
74 (67)	37 (33)

### DOES *NODAL* CONSTITUTE THE *LRA1* GENE HYPOTHESIZED IN THE SSIS MODEL?

**FIGURE 3:**
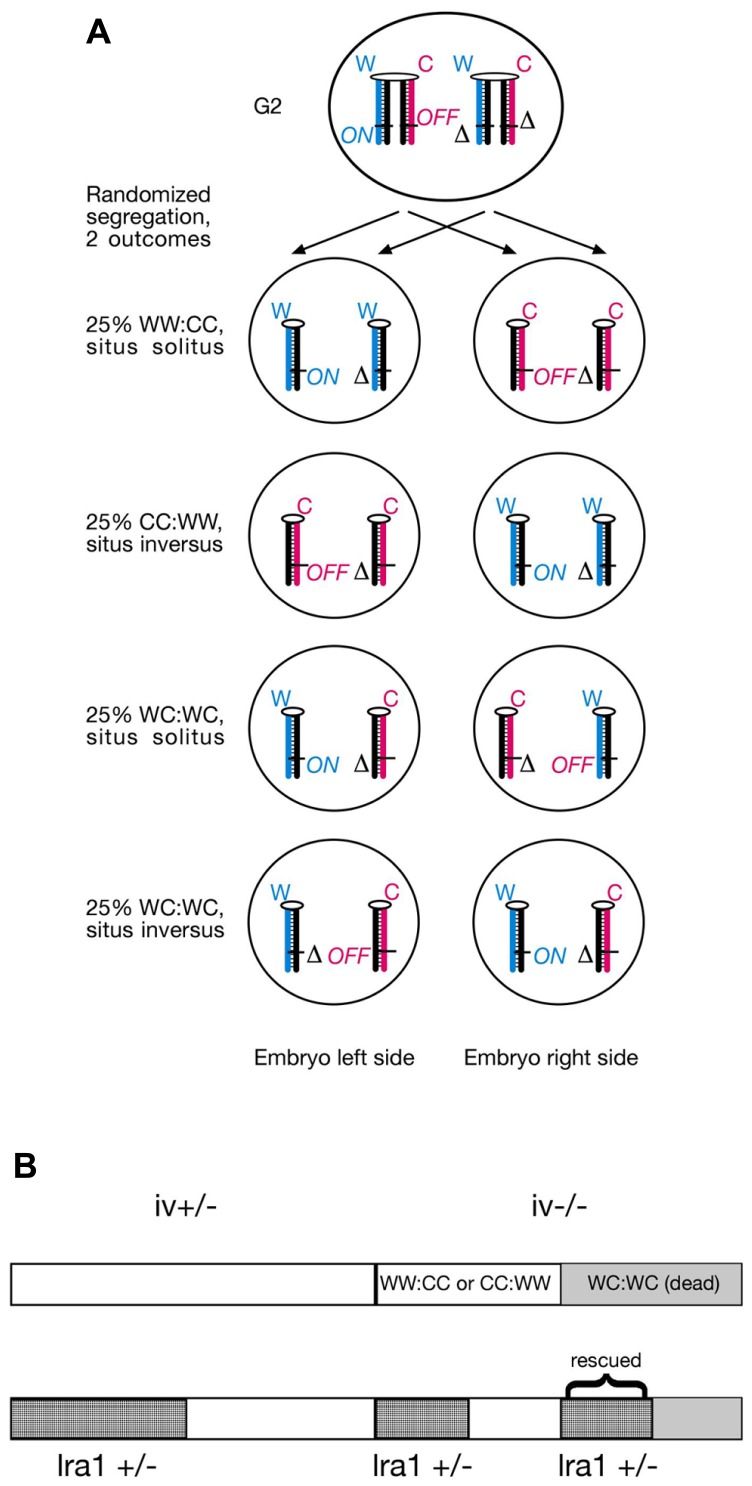
**(A)** *Lra1* heterozygosity is predicted to rescue WC:WC segregants that occur in *lrd* mutants. As illustrated in **Figure 2**, SSIS predicts 50% lethality in *lrd* mutants due to occurrence of WC:WC segregation at 50% incidence. Lethality is due to conflicting (*ON* and *OFF*) *lra1* epialleles in cells that inherited both older Watson and older Crick template strands. However, in compound *lrd* homozygous and *lra1* heterozygous mutant embryos, WC:WC segregants are predicted to survive. This is because *lra1* has only one functional allele, the lethality-causing *ON/OFF* combination in both sister cells described in **Figure 2** cannot be generated. Therefore, a 50:50 distribution of situs solitus and situs inversus animals is expected to develop. Symbols: δ, deletion of *lra1* ( = *Nodal*?); rest of symbols are as described in **Figure 1A**. **(B)** SSIS-predicted ratios of genotypes from an *iv*^+/−^ X *iv*^−/−^ cross (top) and an *iv*^+/−^, *lra1*^+/−^ X *iv*^−/−^, *lra1*^+/+^ cross (bottom). Conventionally 50% offspring is expected to be *lra1*^+/−^. Because WC:WC segregants (gray) are predicted not to die if they are also *lra1*^+/−^,* lra1*^+/−^ animals should be overrepresented in the offspring by a 4:3 ratio. Moreover, *iv*^−/−^ are predicted to occur at a 3:4 ratio as opposed to 1:2 (top), and *lra1*^+/−^, *iv*^−/−^ animals are also predicted to occur at increased rates (2/7).

According to the SSIS model, heterozygosity for the *lra1* gene would prevent embryonic lethality in *iv*^−/−^ embryos because heterotaxia or isomerism would not occur. Consequently, 50% would develop normal organ situs and 50% would develop situs inversus in embryos with *lra1*^+/−^, *iv*^−/−^ genotype (**Figure 3A**). We chose a candidate gene approach, and considered the *Nodal* gene as a likely candidate for the *lra1* gene as it is the gene, other than *iv*, that functions most upstream in the L/R pathway. *Nodal* belongs to the TGF-β family of extracellular signaling molecules and has been shown to be amongst the earliest asymmetrically (left-sided) expressed molecules in a variety of species, ranging from snails to man (Nakamura and Hamada, 2012). Since *Nodal* is essential for mesoderm induction during gastrulation and the mutant embryos die before L/R axis is established (Lowe et al., 2001), its function for L/R axis development is difficult to address by using a conventional null allele. Conditional inactivation of the FoxH1 transcriptional activator in the lateral plate mesoderm causes loss of *Nodal* expression and R/R isomerism (Yamamoto et al., 2003). Likewise, injection of *Nodal*^−/−^ ES cells into wild-type (wt) blastocysts results in development of R/R isomers (Oh and Li, 2002). Thus, *Nodal* is considered to encode “leftness.” If *Nodal* is in fact the *lra1* gene, the 50% lethality of *lrd* homozygous mutant embryos should be suppressed in *Nodal*^+/−^ heterozygotes according to our model (**Figure 3A**).

We determined whether heterozygosity for a null allele of the *Nodal* gene (Lowe et al., 2001) in *iv* crosses affected lethality ratios. Specifically, we determined whether the mutation suppresses 50% lethality of *iv*^−/−^ mice described in **Figure 2**. Because the *Nodal* gene deletion is homozygous lethal, we therefore quantitated viability of only heterozygous animals. We generated several males heterozygous for *iv* and *delta Nodal* mutations, which we set up with *iv*^−/−^, *Nodal*^+/+^ females. Therefore, half of all offspring will be heterozygous for *delta Nodal* allele. In this mating set up, several predictions concerning ratios of expected genotypes are made (**Figure 3B**). Should heterozygous *Nodal* mutation not influence lethality ratios, then 1/3 *iv*^−/−^ mice should be observed, just like the result of the cross described in **Table 2**. Accordingly, 50% will be heterozygous for the *delta Nodal* mutation, and 1/6 (1/3 × 1/2) will be both *iv*^−/−^ and carriers of *delta Nodal*. If heterozygosity for *Nodal* rescues lethality in WC:WC segregants, then only 1/8 (1/2 × 1/2 × 1/2) of initial conceptuses will die, which reduces the total number of observable mice to 7/8. As discussed above, 1/8 of initial mice correspond to 1/7 of observable (live) mice. Three out of seven of live mice will be of *iv*^−/−^ genotype, and 4/7 will be *Nodal*^+/−^, should this mutation suppress lethality in a subgroup of mice destined to die. Moreover, the ratio of *iv*^−/−^ and *Nodal*^+/−^ animals will now be not a simple product of their individual ratios, rather this genotype will be enriched, and is predicted to occur at a 2/7 rate: 1/7 stems from WW:CC (or CC:WW) segregants and 1/7 from rescued WC:WC segregants (**Figure 3B**). The observed result of these crosses is summarized in **Table 3**. Amongst 202 offspring, we found 66 *iv*^−/−^ and 103 *Nodal*^+/−^ animals. Thirty-three mice were both *iv*^−/−^ and *Nodal*^+/−^. These numbers do not support the *Nodal* gene being the hypothetical *lra1* gene. Rather, they show that *lrd* mutation causes 50% lethality in *Nodal* heterozygotes as well.

**Table 3:** Allele frequencies in offspring of *iv*^+/−^* Nodal*^+/−^ × *iv*^−/−^* Nodal*^+/+^ cross.

*n* = 202	Conv. expected	SSIS expected	Observed
*Iv*^−/−^**	1/3 = 67.3	3/7 = 86.6	66
*Nodal*^+/−^	1/2 = 101	4/7 = 115.4	103
*iv*^−/−^ and *Nodal*^+/−^**	1/6 = 33.7	2/7 = 57.7	32

## DISCUSSION

We propose DNA’s chirality and its asymmetric mode of replication as a potential source for installing binary imprints on the chromatin fiber, and selective segregation of thus differentiated sister chromatids to sister cells as a novel and largely uncharacterized molecular mechanism associated with asymmetric cell divisions. The *lrd*-dependent segregation bias of mouse chromosome 7 sister chromatids in mitotic recombination experiments involving ES cells, endoderm cells, and neuroectoderm (Armakolas and Klar, 2007) cells could represent a case for selective sister chromatid segregation. Even though direct evidence for this interpretation is still missing, it led us to further investigate the phenotype of the *lrd* mouse mutant. In our model *lrd* functions to “sort” and selectively segregate sister chromatids based on their replication history in a WW:CC fashion. This L/R symmetry-breaking asymmetric cell division would be oriented along the L/R axis, positional information for it would presumably come from polarized cytoskeleton (Vandenberg and Levin, 2009). Randomization of chromatid segregation in *iv*^−/−^ mice would lead to 25% normal organ situs (WW:CC segregants), 25% situs inversus (CC:WW segregants), and 50% death (WC:WC segregants). The 50 situs solitus : 50 situs inversus distribution in *lrd* mutant live animals has been described in numerous studies, therefore we only focused on assessment of lethality ratios by studying Mendelian inheritance of *lrd* mutant alleles in appropriate genetic crosses. In order to eliminate potential allele-specific or genetic background-specific artifacts, we analyzed two distinct *lrd* null alleles that had been outbred onto mixed backgrounds. Both crosses revealed *lrd* homozygous mutant animals at rates 50% below Mendelian predictions. This result is consistent with our SSIS hypothesis even though it does not provide definitive proof of it. Nearly all studies of mouse laterality stress only the 50% situs solitus: 50% situs inversus phenotype of *iv*^−/−^ mice and ignore the 50% lethality phenotype. Approximately 50% lethality was first noted by Layton (1978) in one of the earliest studies of *iv* mutant crosses. Our results presented here with *iv* confirmed the estimations of Layton (1978) and extended it to the newly made *Lrd-Neo-GFP* allele. One caveat for our SSIS explanation is that the original *iv* allele might be a leaky missense mutation generating the observed effects. It was therefore important to investigate phenotypes of a different allele, which is why we used the second *Lrd-Neo-GFP* allele for our analysis. A third allele was investigated previously, but the analysis was very limited to draw conclusions regarding lethality (Supp et al., 1999). Unfortunately, that allele was not saved (M. Bruckner, personal communication).

We next sought to test another prediction of our model, namely that heterozygosity for the hypothetical *lra1* gene would rescue WC:WC segregants. The rationale therefore is that strand-specific imprinting of *lra1* would lead to conflicting (*ON/OFF*) *lra1* epialleles in both WC:WC sister cells. If one allele of *lra1* is a null allele (due to heterozygosity), then different epialleles cannot be conflicting anymore (**Figure 3A**). We chose a reverse genetics approach and tested the *Nodal* gene as a possible candidate for *lra1*. Analysis of >200 offspring did not show a protective function for *Nodal* heterozygosity in *lrd* mutant animals: therefore, *Nodal* cannot be *lra1*. We did however confirm the 50% lethality phenotype of *iv*^−/−^ genotype, indicating that lethality was not affected by *Nodal* gene dosage.

We have eliminated *Nodal* as a candidate for *lra1*, and its ActR2B receptor can also be disregarded, because a study from En Li’s laboratory (Oh and Li, 2002) recorded situs ambiguous (pulmonary isomerism) in around 40% of *iv*^−/−^* ActR2B*^+/−^ embryos. If *ActR2B* were *lra1*, then the SSIS model predicts occurrence of situs solitus and situs inversus only. The *Nodal* signaling pathway is highly complex and regulated by numerous factors on several levels (Schier, 2009). After Nodal precursor is activated by proprotein convertases and released into extracellular space, Lefty proteins limit its activity via a reaction-diffusion mechanism. Nodal binding to activin receptors is assisted by distinct co-receptors, and sometimes Nodal binds in conjunction with other TGF-β molecules as a heterodimer. Moreover, evidence from zebrafish suggests tight post-transcriptional control of Nodal, Lefty and activin receptor gene expression by microRNAs (Schier, 2009). Given this level of complexity, a reverse genetic screen is unfeasible to tackle the identity of *lra1* gene in the first step of L/R asymmetry development.

**FIGURE 4:**
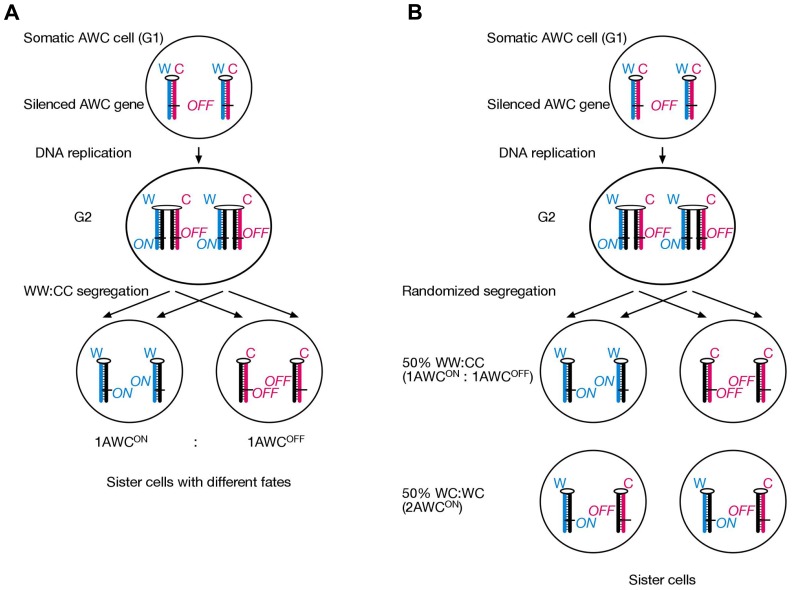
**A finding published by Dr. Michael Levin’s laboratory is interpreted to suggest that an SSIS-like mechanism operates during olfactory neuron asymmetry development in *C. elegans***. **(A)** An AWC precursor cell undergoes asymmetric cell division and selectively segregates epigenetically differentiated sister chromatids containing an AWC master-regulator gene in a WW:CC fashion, such that always a 1AWC^ON^/1AWC^OFF^ olfactory cell pair develops in each worm. **(B)** Embryos transgenic for mutated (but not wild-type) tubulin developed either 1AWC^ON^/1AWC^OFF^ or 2AWC^ON^ olfactory neuron cells at a roughly 50–50 frequency. We explain this result by the SSIS model due to randomized chromatid segregation during the critical AWC^ON^/AWC^OFF^ neuron generating cell division due to the tubulin mutation.

Interestingly, two recent studies have suggested that the nematode *C. elegans* employs SSIS mechanism during Neuronal asymmetry development. A study from Michael Levin’s research group provides genetic support that an SSIS-type asymmetric cell division operates in olfactory neuron development, although the evidence has not been interpreted as such by the authors. The Levin laboratory has a long-standing interest in vertebrate L/R axis development, and has highlighted the role of the cytoskeleton in cellular polarization for years (Aw and Levin, 2009; Vandenberg and Levin, 2009,^2010^). It had come to the authors’ attention that *Arabidopsis* mutants affecting radial flower symmetry were mapped to alpha-tubulin and a gamma-tubulin associated protein (Lobikin et al., 2012). Remarkably, introducing the same alpha-tubulin mutation into *Xenopus* 1-cell embryos resulted in development of heterotaxia, and in cultured human HL-60 cells it disturbed the leftward bias (with respect to the nucleus-centrosome axis) of pseudopodia protrusion. In addition, *C. elegans* “AWC” olfactory neural asymmetry was also affected by mutating a tubulin homolog (TBA-9, 75% amino acid identity with Arabidopsis alpha-tubulin) at two conserved amino acids. In wt worms, the AWC neuron is in the “ON” state (AWC^ON^) on one body side and in the “OFF” state (AWC^OFF^) on the other body side; sidedness is stochastic (Chang et al., 2011). This developmental asymmetry can be visualized by introducing the “str-2p::GFP” fluorescent GFP construct in the genome. Importantly, chromosomal integration site of the GFP transgene is irrelevant for faithful AWC^ON^ versus AWC^OFF^ discrimination, indicating that the cause for asymmetric GFP expression acts in trans for the transgene. The authors chose this model system for body asymmetry development studies, because the AWC^ON^ and AWC^OFF^ cells show cytoskeletal polarization and asymmetric calcium signaling, which is sensitive to the microtubule depolymerizing drug nocodazole (Chang et al., 2011). Overexpression of wt TBA-9 tubulin in transgenic worms causes only mild laterality defects, with 82% of worms displaying the normal 1AWC^ON^/1AWC^OFF^ phenotype. Overexpression of mutant TBA-9, in contrast, results in 42% normal 1AWC^ON^/1AWC^OFF^ and 45% novel 2AWC^ON^ “heterotaxic” phenotype. This roughly 50:50 distribution is consistent with a SSIS mechanism operating in the mother AWC cell (**Figure 4**). We hypothesize that one daughter inherits normally two AWC^ON^ epialleles and the other two AWC^OFF^ epialleles are inherited by the other daughter cell (WW:CC segregation). Unlike our SSIS model for mouse L/R axis development, this asymmetric distribution of sister chromatids in the worm occurs irrespective of the L/R body axis. We propose that introduction of mutated tubulin renders the AWC cell’s cytoskeleton unable to direct selective chromatid segregation in mitosis, hence a novel WC:WC segregation results at 50% frequency. A simple explanation for 2AWC^ON^ phenotype in WC:WC segregants would be dominance of the AWC^ON^ over the AWC^OFF^ epiallele.

A second study implicating an SSIS-like asymmetric cell division in *C. elegans* neuronal asymmetry development has been recently published by Horvitz/Stillman laboratories (Nakano et al., 2011). Here, a GFP-reporter screen served to isolate mutants that changed the paired asymmetric MI motor neuron/e3D epidermal cell pair to a symmetrical e3D cell pair on both sides of the brain. Positional cloning identified a gain-of-function mutation in a *histone H3* gene that deleted its last 11 amino acids, thereby impairing the ability to form H3/H4 tetramers during chromatin assembly. Likewise, RNAi against chromatin assembly factor 1 (CAF-1) or PCNA pheno-copied the H3 mutant. The authors suggest that newly lagging strand synthesized DNA contains elevated levels of PCNA and associated CAF-1 containing histone chaperone complex, which deposits higher nucleosome density. This could represent an epigenetic imprint in itself, or serve to nucleate covalent chromatin modifications. The latter seems somewhat more likely, since the epigenetic imprint is transmitted through several mitoses, as it is the MI/e3D great-great-grandmother cell that directs development of distinct cell fates three cell divisions later on. Like in our SSIS model, selective segregation of epigenetically differentiated sister chromatids is an integral part of the authors’ model. However, neither direct nor indirect evidence is presented. Because genetic evidence suggests that mutated tubulin (Lobikin et al., 2012) randomizes (the normally selective) chromatid segregation during an AWC^ON^/AWC^OFF^ olfactory neuron asymmetry generating cell division, we propose to test whether mutated TBA-9 also affects the MI/e3D neuronal asymmetry.

The SSIS model is conceptually based on three aspects: (i) differential chromatin imprinting during inherently lagging versus leading strand replication, (ii) one or several genes who’s expression is affected by this imprint, and (iii) a segregator that identifies and “sorts” epigenetically differentiated sister chromatids by operating at sister centromeres in mitosis. We have presented genetic evidence for (iii), namely that *lrd* acts as a segregator in a L/R axis defining cell division in mouse. In contrast, Nakano et al. (2011) have provided evidence for (i), but have not identified the segregator. If the segregator can be identified, *C. elegans* will be excellently suited to use forward genetics to identify the gene or set of genes (ii) that are imprinted and selectively segregated, as outlined in our *iv*^−/−^, *Nodal*^+/−^ breeding experiment.

We have highlighted two studies that support a SSIS-type mechanism in the development of neuronal asymmetries in *C. elegans*. Based on genetics of psychosis development in human carriers of balanced chromosome 11 translocations, we have previously proposed that a similar mechanism may operate during human brain lateralization (Klar, 2004). Analogous to our model for body laterality development, brain laterality development would also initiate with a single critical asymmetric cell division, where chromosome 11 sister chromatids are selectively segregated in a WW:CC fashion. If one chromosome 11 homolog is fused to the centromere of another chromosome not undergoing selective mitotic segregation, then WW:CC and WC:WC segregation for chromsome 11 are expected to occur at equal frequencies. Fifty per cent incidence of psychosis development in four different families with balanced chromosome 11 translocations support our hypothesis (Singh and Klar, 2007).

Whether asymmetric cell divisions elsewhere during normal tissue homeostasis employ a SSIS mechanism remains to be determined. If they do exist, then somatic chromosomal translocations could potentially randomize these asymmetric cell divisions and initiate tumorigenesis. An example would be a resting tissue stem cell that only enters the cell cycle upon tissue injury. It asymmetrically divides to produce a rapidly-proliferating transiently amplifying stem cell. This cellular asymmetry development would be controlled by asymmetric segregation of cytoplasmic determinants, but also by WW:CC segregation of epigenetically differentiated sister chromatids, where cell cycle promoting genes remain silenced in the mother cell, but poised for expression in the transiently amplifying daughter cell. A chromosomal translocation involving the chromosome undergoing selective segregation in the tissue stem cell could therefore change the WW:CC pattern to a WC:WC pattern. As a result, the resting tissue stem cell would acquire proliferative capacities of the transiently amplifying stem cell, leading to neoplasia. Additional oncogenic mutations will eventually render this cell growth cancerous. Despite this example being rather simplistic, it should be appreciated that genes controlling asymmetric cell division are increasingly recognized as tumor suppressors. Drosophila *brat* and *prospero* mutants, for example, fail to undergo asymmetric neuroblast cell divisions, and develop larval brain tumors (Betschinger et al., 2006). We suggest that somatic chromosomal translocations in tissue stem cells could affect biased segregation of sister chromatids, and change strictly asymmetrically dividing stem cells to stem cells that undergo symmetrical cell divisions in terms of epigenetic imprints on differentiated sister chromatids distal to the translocation breakpoint.

Curiously, a 1992 study published in *The Lancet* (Sandson et al., 1992) found a correlation of abberrant brain laterality development and breast cancer. Right-handed breast cancer patients and healthy controls were subjected to computer tomographic brain scans. Eighty-two per cent of control subjects showed left hemispheric dominance, whereas in the breast cancer group this number was reduced to 51%. Although this study should be cautiously interpreted until replicated elsewhere, it certainly suggests that brain laterality- and breast cancer-development share a common genetic pathway (Klar, 2011). We suggest that this pathway controls asymmetric cell divisions during embryonic brain development, and during cell turnover in the lactiferous duct upon periodic hormonal growth stimulation. Hence, improving our understanding of vertebrate laterality development could eventually impact on cancer prevention and treatment.

Taken together, 50% lethality phenotype in the *lrd* mouse mutant supports predictions made by the SSIS model for laterality development. Here, *lrd* is part of a cellular mechanism that selectively segregates epigenetically differentiated sister chromatids concerning their replication history with respect to a cytoskeleton-based early L/R axis (Klar, 2008; Vandenberg and Levin, 2010; Lobikin et al., 2012). The overwhelming majority of studies on *lrd* in the mouse have focused on its role in conferring nodal cilia motility. This is understandable, since genetics of spontaneous and targeted mouse mutations affecting laterality development have generally pointed to a central role for motile nodal cilia. Moreover, the earliest known molecular L/R asymmetries appear after node formation in the mouse. In chicken and *Xenopus*, in contrast, earlier asymmetries involving Gap-junctional communication (Levin and Mercola, 1999), H^+^/K^+^ ATPase activity (Levin et al., 2002), and serotonin signaling (Fukumoto et al., 2005) have been identified. As many of the studies on earlier asymmetry determinants in frogs and chicken involved embryo-exposure to pharmacological inhibitors, mouse embryo-culture protocols will need to vastly improve until replication can even be considered. In this regard it is noteworthy that one of the leading laboratories for mouse embryo *in vitro* culture has recently tested the relationship of nodal cilia emitted force and asymmetry development in several mouse mutants affecting cilia biogenesis and motility (Shinohara et al., 2012). Surprisingly, the authors found that as few as two motile nodal cilia were sufficient to break bilateral symmetry. These data are rather compatible with the “2-cilia hypothesis,” which was initially postulated by the Hirokawa, Brueckner, and Tabin laboratories (Okada et al., 1999; McGrath et al., 2003; Tabin and Vogan, 2003). Here, mechanical force exerted by beating nodal cilia is read out by mechanosensory cilia, which are associated with polycystin-2 (a calcium release channel) to induce left-sided calcium signaling. Hence loss of *polycystin-2* is predicted to ablate calcium and Nodal signaling altogether. Pennekamp et al. (2002) indeed found loss of *Nodal* expression in the majority of *polycystin-2* deficient embryos, however the *Nodal* downstream target *Pitx2* showed bilateral expression. Clearly how body laterality is initially developed, whether in visceral organs or in the nervous system, remains controversial thus far. Further work is needed to differentiate if any of the prevailing hypotheses can satisfactorily explain body laterality development. The SSIS model is simple to understand in that an asymmetric cell division constitutes the root cause of development. In this model, developmental decisions are made through particulate matter consisting of *ON/OFF* epigenetic states of gene expression of developmentally important gene(s). Thus, in addition to acting as genetic material, DNA strands can provide the basis for evolution, cancer and development (Furusawa, 2011).

## Conflict of Interest Statement

The authors declare that the research was conducted in the absence of any commercial or financial relationships that could be construed as a potential conflict of interest.
